# Methods for Detecting Abnormal Ventilation in Children - the Case Study of 13-Years old Pitt-Hopkins Girl

**DOI:** 10.1177/2329048X231151361

**Published:** 2023-02-20

**Authors:** Pekka Nokelainen, Jose-Maria Perez-Macias, Sari-Leena Himanen, Anna Hakala, Mirja Tenhunen

**Affiliations:** 1 Department of Clinical Neurophysiology, Medical Imaging Centre, Pirkanmaa Hospital District, Tampere, Finland; 2 Outpatient Clinic for Patients with Intellectual Disability, Pirkanmaa Hospital District, Tampere, Finland; 3 Faculty of Medicine and Health Technology, 7840 Tampere University, Tampere, Finland; 4 NeuroEvent Labs, Tampere, Finland; 5 Department of Medical Physics, 60670 Tampere University Hospital, Medical Imaging Centre, Pirkanmaa Hospital District, Tampere, Finland

**Keywords:** sleep, sleep-disordered breathing, respiratory effort, Pitt-Hopkins, Emfit sensor, NEL seizure detection

## Abstract

We present contactless technology measuring abnormal ventilation and compare it with polysomnography (PSG). A 13-years old girl with Pitt-Hopkins syndrome presented hyperpnoea periods with apneic spells. The PSG was conducted simultaneously with Emfit movement sensor (Emfit, Finland) and video camera with depth sensor (NEL, Finland). The respiratory efforts from PSG, Emfit sensor, and NEL were compared. In addition, we measured daytime breathing with tracheal microphone (PneaVox,France). The aim was to deepen the knowledge of daytime hyperpnoea periods and ensure that no upper airway obstruction was present during sleep. The signs of upper airway obstruction were not detected despite of minor sleep time. Monitoring respiratory effort with PSG is demanding in all patient groups. The used unobtrusive methods were capable to reveal breathing frequency and hyperpnoea periods. Every day diagnostics need technology like this for monitoring vital signs at hospital wards and at home from subjects with disabilities and co-operation difficulties.

## Introduction

Detecting respiratory effort and hypoventilation in children is often difficult and demanding, more so in children with disabilities. Polygraphy recordings with several bio signals are challenging and need monitoring and laborious interventions by skilled personnel. The Gold standard for assessing respiratory effort is esophageal pressure measurement (pESO). It detects changes of pleural pressure produced by work of inspiratory muscles but requires use of trans-nasal pressure sensor catheter. Indirect methods such as inductive abdominal/thoracic belt sensors and surface electromyography (EMG) of respiratory muscles have been recommended to use in clinical polysomnography (PSG) studies.^1,2^ The thoracic volume which are estimated from cross-sectional area changes by belts, do not reflect muscle activity when upper airway impedance changes during respiration and neither increased EMG activity is reliable measure for respiratory effort.^3,4^ The surface electrodes detach easily and are mainly used in neonatal recordings. These techniques tend to interfere with subject's sleep and normal activity.

In this study, we present alternative methods for quantifying disordered breathing in cases where the use of conventional sensors is difficult. Our patient has Pitt-Hopkins syndrome, where sleep difficulties are quite common and almost half of the patients have abnormal breathing pattern during wakefulness, consisting of repeating hyperpnoea periods with apneas in between.^5^

**Figure 1.**
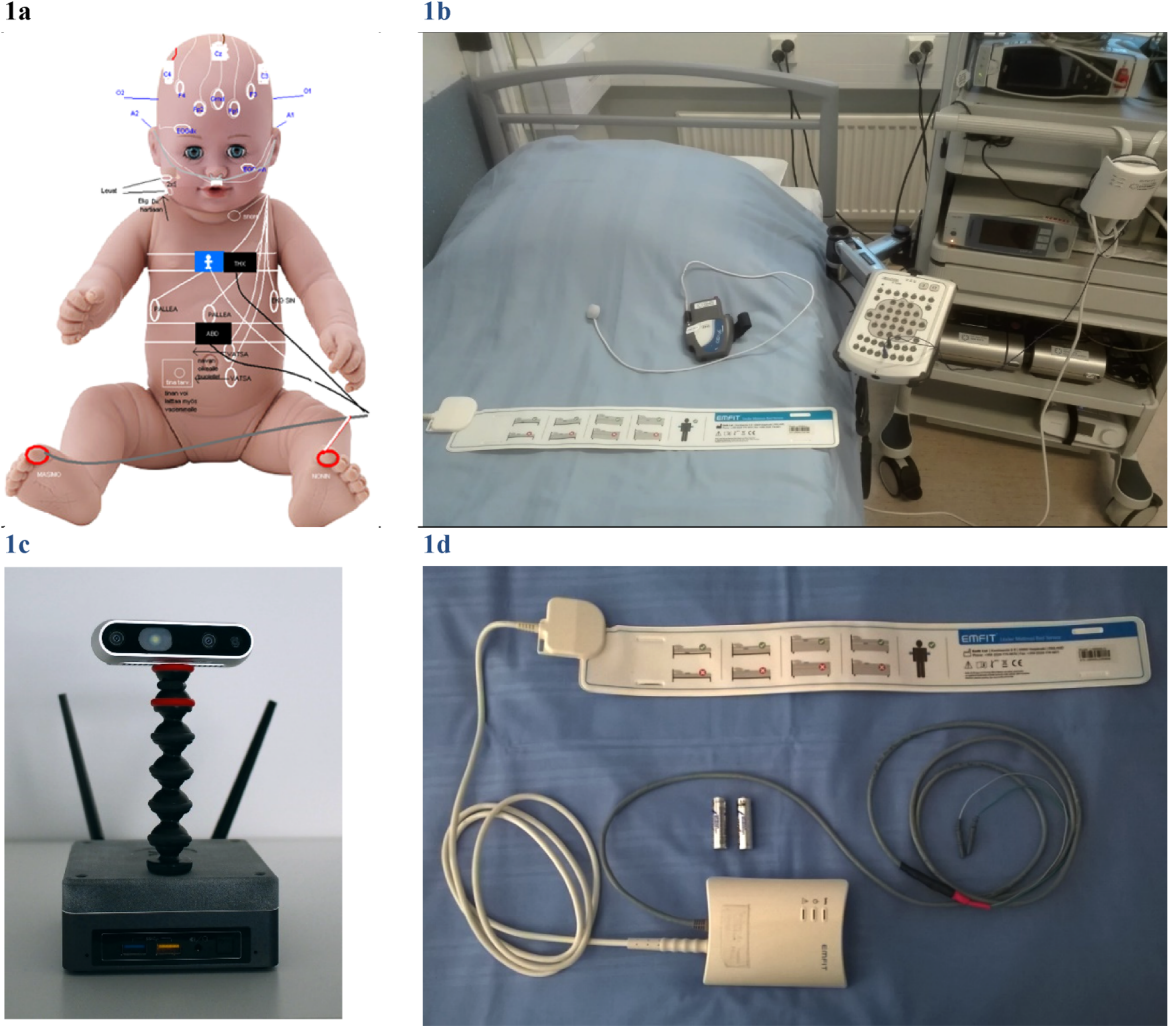
Polysomnography acquisition was done with external devices. (**a)** ‘A little patient’ with extended PSG equipment and accessories. **(b)** Emfit mattress sheet which can be connected to Embla N7000 bedside unit via bipolar inputs. The grey Cidelec CID-Lxe device with PneaVoX sound sensor lay on the bed. (**c**) Depth sensor station NEL. (**d**) Emfit sensor is placed under a foam mattress and subject's thoracic area.

Our aim is to evaluate and characterize the patient's nocturnal sleep and breathing. At the same we test detection of breathing with depth sensor attached to video camera (NEL, Figure 1c). The technique is previously used in epilepsy monitoring^6–9^ but video-based algorithm for respiratory detection is developed also.^10,11^ In addition, we use the electromechanical film transducer (Emfit, Figure 1d) mattress, which reveals respiratory efforts as high frequency spiking phenomenon in Emfit signal.^12,13^

## Materials and Methods

### Patient

A 13-years old girl (length 135 cm, weight 32 kg, BMl 17,6) suffering from Pitt-Hopkins syndrome (PTHS) was studied in the Sleep Laboratory. The reason for referral was periods of hyperpnoea and apneic spells at wakefulness with a suspicion of additional breathing abnormalities during sleep. The clinical characteristics of PTHS include microcephaly, small face, narrow airways, psychomotor delay, and intellectual disability. It is caused by molecular variants of the TCF4, which is a transcription factor involved in neuronal differentiation during embryonal development. This unusual breathing pattern becomes evident between 3–7 years of age, but with large variation sometimes it is seen much later, in early adulthood. Typical pattern during wakefulness shows firstly few minutes of hyperpnoea, followed by an apnea with complete stop of breathing, which can be long enough to induce cyanosis. About half of the PTHS patients are thought to show this pattern of hyperventilation with or without the apnea. Despite being disturbing to witnesses, are mostly kept harmless.^14–16^ Medication is however sometimes used. Due to swallowing a lot of air during these spells, patients show problems of indigestion and sometimes even frequent pneumonia due to aspiration. During the sleep the breathing is thought to be normal. Due to rarity of the syndrome good quality sleep recordings are difficult to obtain. There is also need for ventilation monitoring to exclude possibility of obstructive character of breathing. During the clinical examination child neurologist had noticed our patients’ typical microcephaly features, narrow upper airways, delayed cognitive development, and difficulties with co-operation. The patient's mother gave written informed consent to use external devices as the part of clinical polysomnography and to use data as anonymized in further analysis. The study was approved by the ethics committee of Tampere University Hospital.

### Daytime Respiratory Monitoring (Cidelec  +  Emfit  +  NEL)

**Figure 2.**
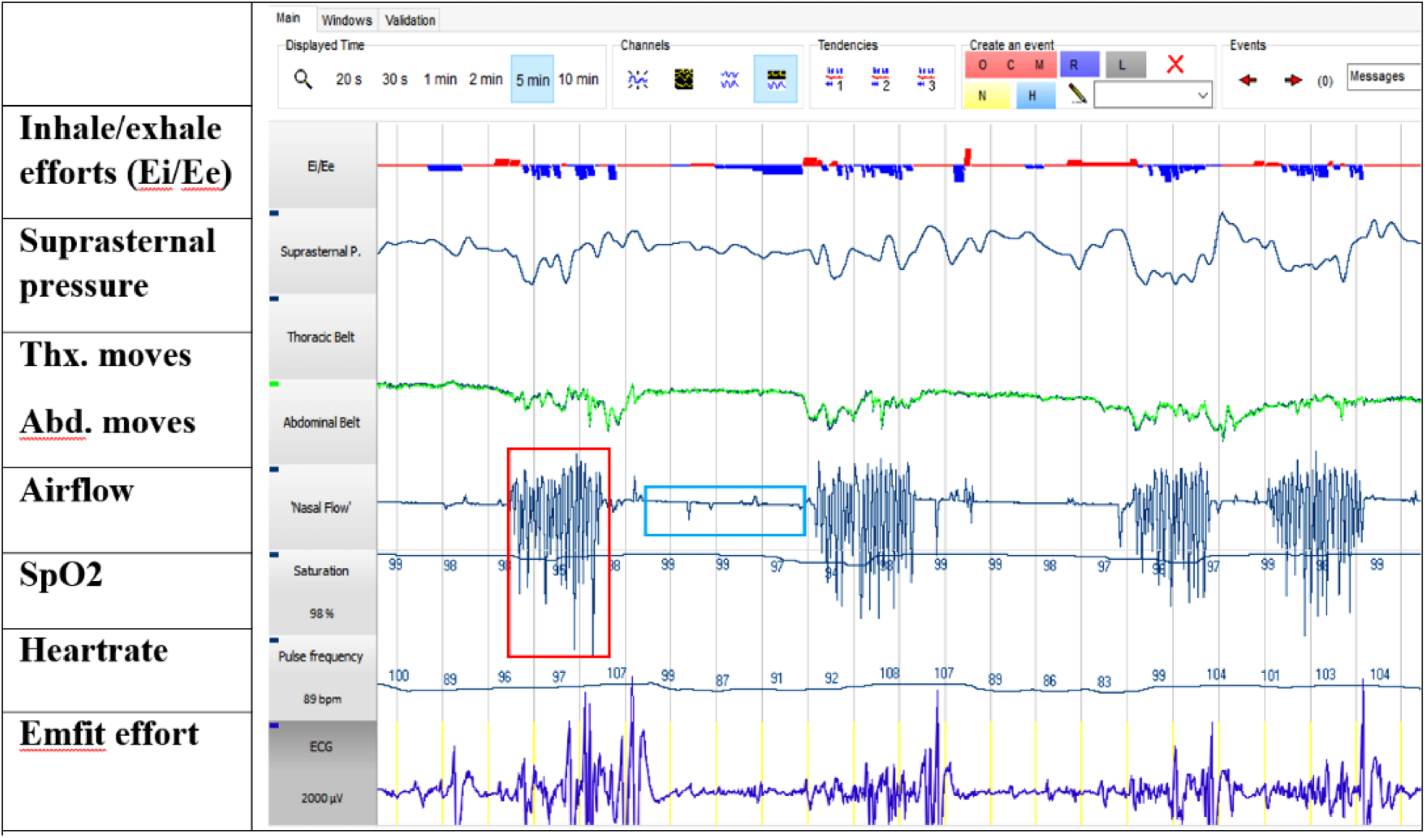
Screenshot (5 min) of daytime Cidelec analysis software showing the hyperbreathing periods emphasized in Airflow channel (4 periods, one with red square). Long apneas occurs between those gasping periods (light blue square). Suprasternal pressure signal measured with tracheal microphone was used to calculate respiratory effort. The Ei/Ee trace visualize that expiration (Ee blue color, down) is emphasized over inspiration (Ei red color, upfords) during hyperpnoea. In addition, the increased breathing efforts are seen in Emfit mattress sensor.

To evaluate the details of patient's hyperventilation periods in wakefullness, we made a daytime breathing monitoring with Cidelec polysomnography device CID-LXe. It included PneaVox sensor for pharyngeal pressure and sound monitoring (Cidelec, France) (Figure 1b). The analysis is based on acoustic and pressure variation measures, which show upper airways resistance changes and the ratio of inspiratory effort (Ei) over expiratory effort (Ee) is calculated. In addition, Emfit mattress acquisition (Emfit ltd, Finland) (Figure 1d) and video monitoring with depth sensor (NEL, Neuro Event Labs, Finland) (Figure 1c) was also performed and triggered to Cidelec PSG recording. The measured signals are presented in Figure 2.

### Nighttime Monitoring, Polysomnography PSG

Overnight PSG was performed with Embla N7000 device (Embla, Natus Medical Inc., USA) and Remlogic software (Embla Systems LLC, USA). The recording consisted of eight EEG derivations (Fp1-M2, Fp2-M1, F3-M2, F4-M1, C3-M2, C4-M1, O1-M2, O2-M1), two electro-oculography channels (EOGdx and EOGsin), three electromyogram (EMG) channels (submental and both legs) and electrocardiogram (ECG). Airflow and respiratory movements were monitored with a thermistor and a nasal pressure transducer and thoracic and abdominal inductive belts. EMG signals from diaphragm and abdomen were installed to estimate breathing effort. Pulse and oxygen saturation were measured by PSG device integrated pulseoximeter (Nonin Medical Inc, USA) and position with activity sensor (Figure 1a). In addition, simultaneous Emfit mattress, video monitoring and end-tidal carbon dioxide (etCO2) measurement (CAP10, Germany) were performed. A sampling rate of 2 Hz was used for the pulse oximetry (SpO2 and pulse rate), 10 Hz for respiratory movements, 500 Hz for ECG, and 200 Hz for the other signals.

## Results

The breathing in daytime polygraphy was mostly periodic, consisting of the hyperpnoea episodes lasting up to 25-30 s with intermittent apneas lasting 50–60 s (Figure 2). During hyperpnoea, expiratory breathing effort predominated over inspiratory effort.

**Figure 3.**
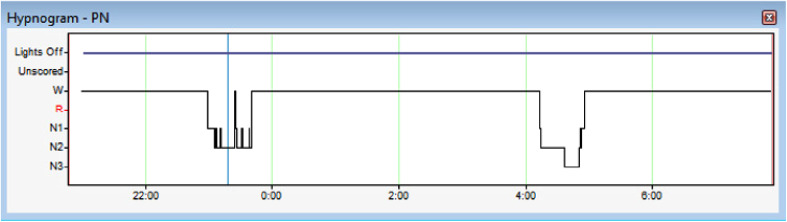
The amount of sleep in the laboratory night was minor and there was no REM sleep at all.

The clinical neurophysiologist analyzed and scored the nocturnal PSG manually. PSG analysis gave the following parameters: Time in bed (TIB) 653 min, total sleep time (TST) 82,5 min, sleep efficiency index (TST/TIB) only 12,6%. Sleep stages (percentage from TST): N1 18,2% (15 min), N2 65,5% (54 min), N3 16,3% (13,5 min), REM-sleep 0%. Sleep latency was 1195 min. WASO was 5705 min. Arousal index 25,5/h. In addition, few single EEG spikes were recognized from both hemispheres. The hypnogram is presented in Figure 3. The first sleep episode was fragmented consisting only N1 and N2 sleep. The second period included N2 and N3 sleep. The patient's mother told that at home in familiar environment the patient usually sleeps much better.

Apnea-hypopnea index (AHI) was 0/h. There were no obstructive, central or mixed apneas neither hypopneas during sleep. The patient slept only in supine position. ODI3 was 2,2/h. SpO2 minimum was 86%, SpO2 in average was 94,3%. Pulse in average 99 BPM, range 36–150 BPM.

**Figure 4.**
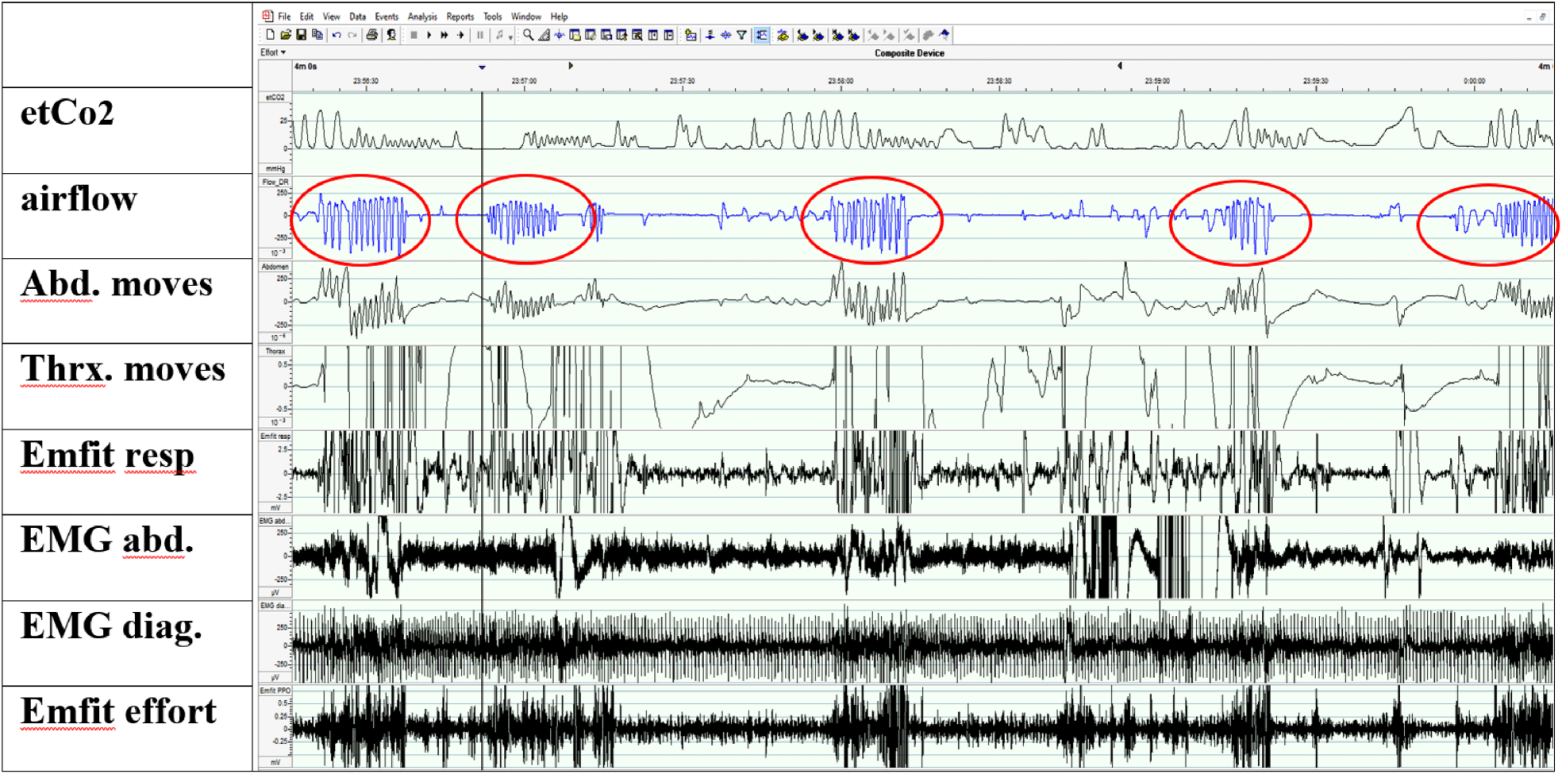
Wakefulness period (4 min) in nighttime polysomnography showing the hyperpnoea periods (red circles) followed by apneic spells. EtCo2 was measured by capnometry. Airflow was measured from the nasal pressure sensor. Breathing movements were detected by inductive belt traces (Abd and Thx moves) and by Emfit mattress (Emfit resp). Breathing efforts were measured by surface EMG electrodes from abdomen and diaphragm muscles (EMG abd, EMG diag). Emfit channel with filtration 6–16 Hz shows the typical spiking phenomenon implying increased respiratory effort.

The breathing in wakefulness before falling asleep varied remarkably (Figure 4) consisting of alternating hyperpnoea episodes and central apneas (breath holds). Respiratory frequency during normal breathing in wakefulness was about 22/min. End-tidal carbon dioxide (etCo2) values increased to ad 39–40 mm Hg (5.2-5.3 kPa) in the first sleep period. During the second sleep period the highest etCo2 values were ad 41 mm Hg (5.5 kPa). Oxygen saturation values varied between 93% and 96%.

**Figure 5.**
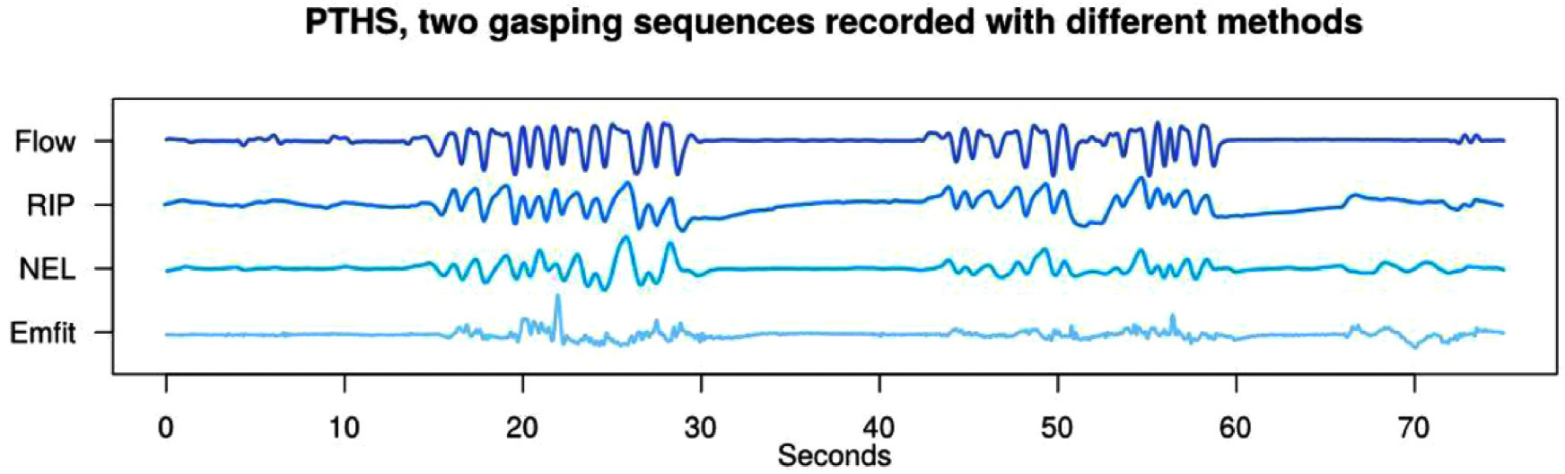
Hyperpnoea periods analyzed from conventional PSG (Flow and RIP), video and depth sensor (NEL) and Emfit mattress. Hyperpnoea periods and following apneas are revealed by all sensors. Flow  =  airflow by nasal prongs, RIP  =  respiratory belt, NEL  =  video and depth sensor, Emfit  =  Emfit mattress signal.

The researcher from Neuroevent Lab analyzed the NEL video/depth sensor signal and produced time sync traces of different respiratory signals. Figure 5 presents that the hyperonoea in PTHS can be detected noninvasively with both the NEL system and Emfit mattress.

## Discussion

Our aim to evaluate the nocturnal sleep of the patient suffering of Pitt-Hopkins syndrome succeeded only partially. Sleep laboratory environment and multiple sensors disturbed the patient and sleep time remained minor. Sleep macrostructure was abnormal, with no REM- sleep. During short sleep, however, there were no signs of upper airway obstruction even if the upper airways were narrowed. The breathing during REM sleep could not be evaluated since the patient had no REM sleep at all. Unfortunately, night in the laboratory was worse than at home and the problems caused by sleeping environment hinders reliable conclusions.

During both daytime and nighttime wakefulness, the typical hyperpnoea periods with apneic spells were detected. EtCo2 levels were low in wakefulness (4.5-4.7 kPa) and collapsed after the hyperpnoea periods, which supposedly is responsible for the long apneas. The pathophysiology behind the pattern has remained unclear.^5^ Interestingly, during hyperpnoea the expiratory breathing effort prevailed. Abnormal autonomic nerve system and respiratory control are thought to be responsible for the unusual breathing pattern, but the expiratory effort increase is not described previously.

In addition to the conventional PSG, abnormal breathing during wakefulness was successfully recorded with two nonobtrusive devices; the Emfit mattress sensor and the NEL depth sensor. The validity of Emfit mattress diagnostics of breathing disorders has tested against the pESO, the Gold standard of measuring respiratory effort.^13^ Contactless mattress suits well for long term monitoring at home or hospital wards. For now, unfortunately, automatic analysis of Emfit is focused on monitoring recovery and readiness of athletes and healthy customers, and respiratory analysis is performed manually. This hinders its usefulness in respiratory diagnostic.

Video recordings with depth sensors (NEL) can be used for real-time monitoring of breathing parameters like frequency and amplitude. The method is an interesting area of focus when developing future contactless diagnostic techniques. Artificial intelligence algorithms based on video and sound are used to detect motor epileptic seizures and improve the accuracy of subjective seizure diaries.^9^ Even the pajamas and duvets will absorb part of the movement intensity, we show, that respiratory movements can be detected by NEL depth sensor. Currently the signals need off-line analysis handling and processing before the data is ready to visual analysis and interpretation. Therefore, there is need for further developing of contactless or wearable sensors, which combines benefits of all available noninvasive techniques and is capable to provide suitable parameters automatically.

Children with disabilities is a demanding patient group for diagnostic wards. They often need extra hands and family to help co-operation. Detaching electrodes and signal artifacts can easily impair diagnostic accuracy. The presented new contactless technology can give valuable help in respiratory diagnostic.

## Conclusion

This paper describes unified contact-free, low-cost, noninvasive techniques for detection of abnormal hyperpnoea events in child with PTHS syndrome. The diagnostic performance of the detection systems was compared with the PSG. The results suggest that it is possible to identify both hyperpnoea and apnea events with contactless devices. These innovative detection systems could represent a timely, user-friendly and 24/7 tool to be used for example in the neonatal intensive care units, casualty, and emergency units or at the patient's home to monitor different symptoms.
